# Supplementary figures and images for: Genetic variation at the Cyp6m2 putative insecticide resistance locus in Anopheles gambiae and Anopheles coluzzii
Source: Malar J. 2021 May 25;20:234. doi: 10.1186/s12936-021-03757-4 (PMC8146665; doi:10.1186/s12936-021-03757-4)

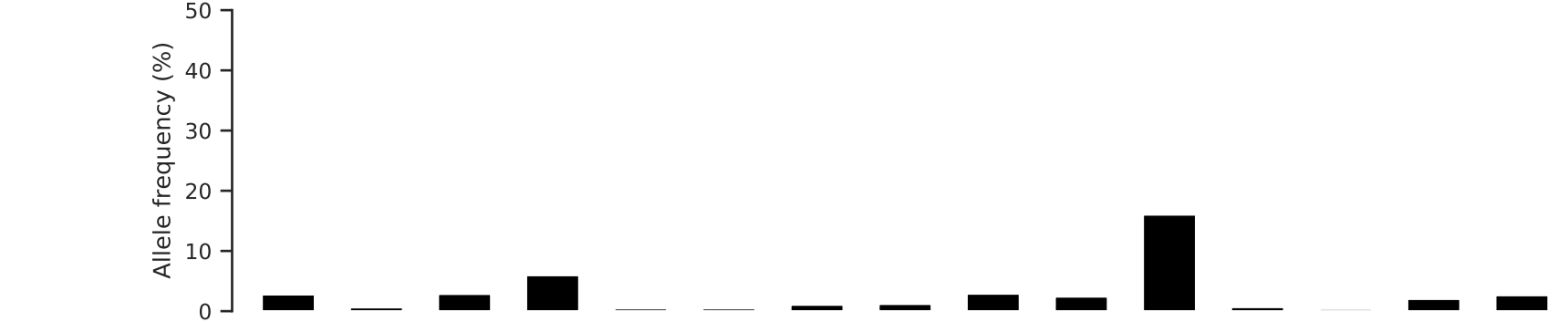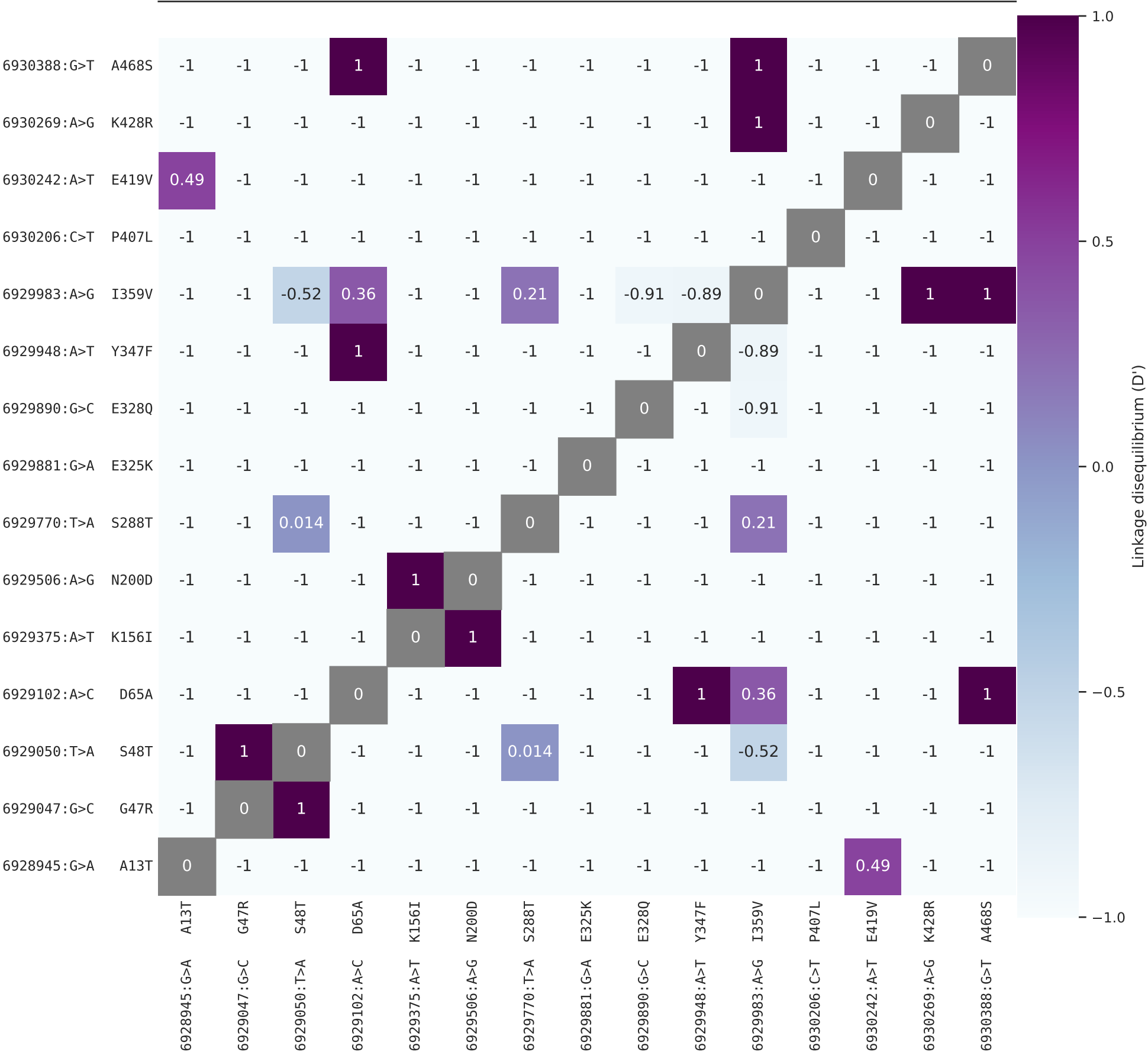

Supplement: Supplementary file 3 — Additional file 3: Fig. S1. Linkage disequilibrium (D′) between non-synonymous variants. A value of 1 shows perfect linkage between the alleles. A value of − 1 shows that the alleles are never found conjointly. The bar plot indicates allele frequencies within the Ag1000G phase 2 cohort. [file 12936_2021_3757_MOESM3_ESM.pdf]

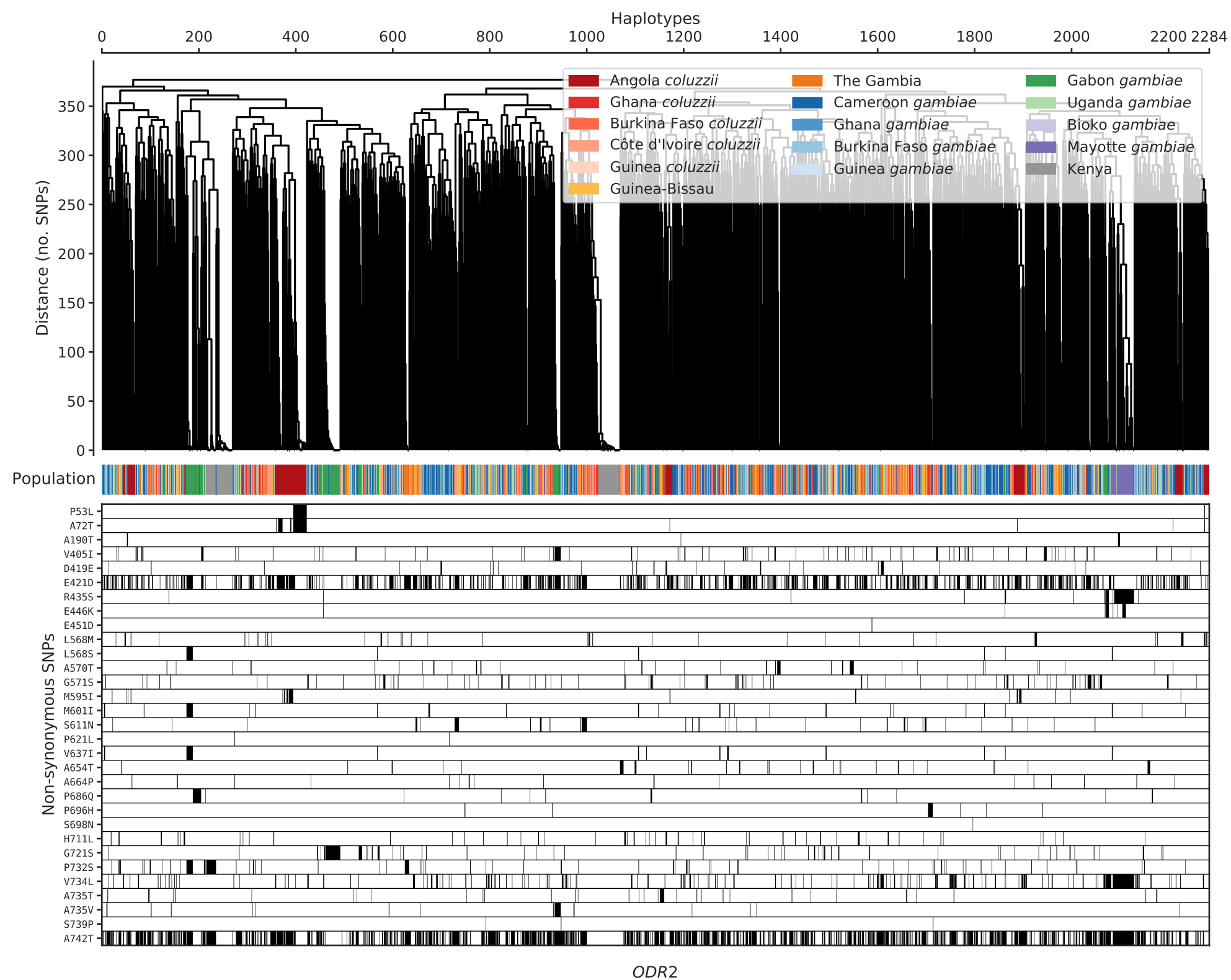

Supplement: Supplementary file 4 — Additional file 4: Fig. S2. Hierarchical clustering and missense mutations for ODR2. Top: a dendrogram showing hierarchical clustering of haplotypes across the ODR2 gene. The gene is located at position 7,059,422 to 7,119,244: 128,875 bases downstream of Cyp6m2. The colour bar indicates the population of origin for each haplotype. Bottom: high frequency (> 5%) alleles identified within each haplotype (white = reference allele; black = alternative allele). [file 12936_2021_3757_MOESM4_ESM.pdf]

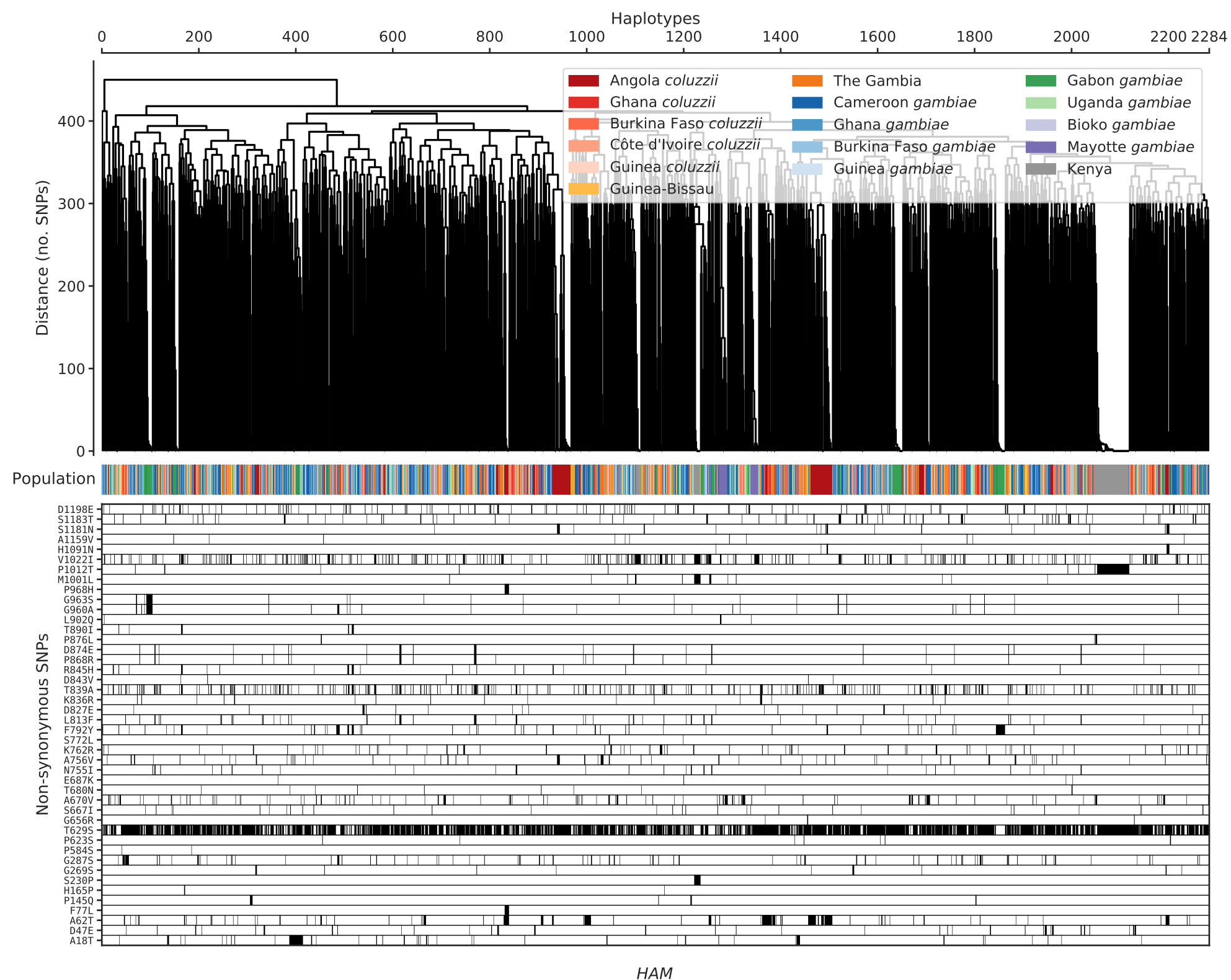

Supplement: Supplementary file 5 — Additional file 5: Fig. S3. Hierarchical clustering and missense mutations for HAM. Top: a dendrogram showing hierarchical clustering of haplotypes across the HAM gene. The gene is located at position 7,435,306 to 7,485,012: 504,759 bases downstream of Cyp6m2. The colour bar indicates the population of origin for each haplotype. Bottom: high frequency (> 5%) alleles identified within each haplotype (white = reference allele; black = alternative allele). [file 12936_2021_3757_MOESM5_ESM.pdf]

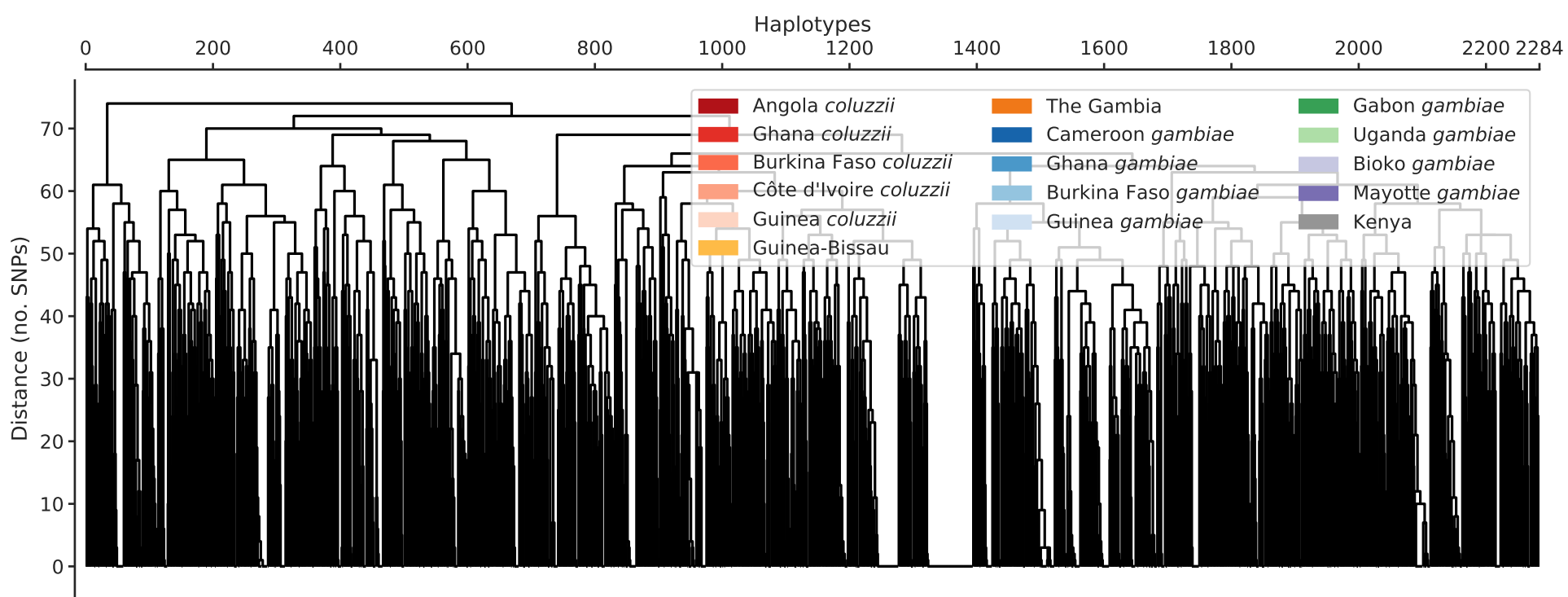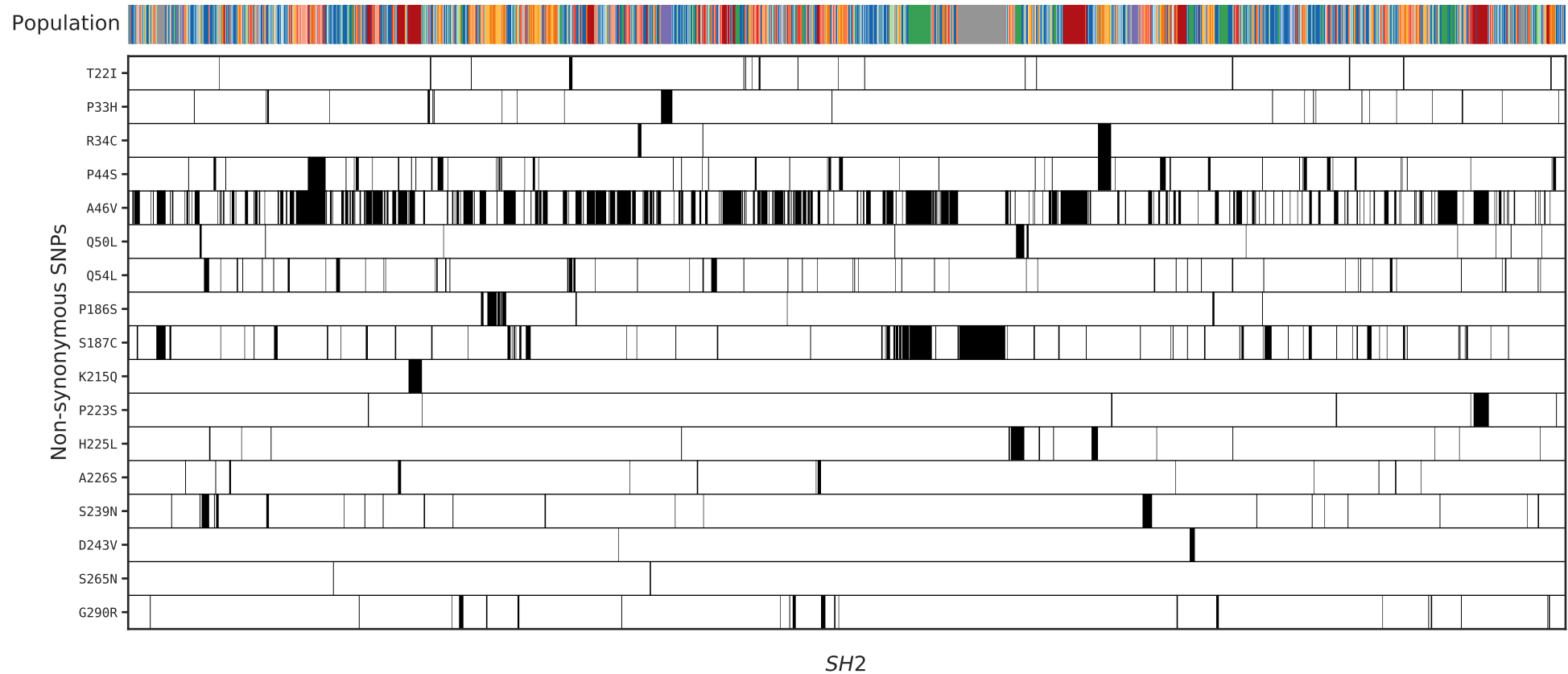

Supplement: Supplementary file 6 — Additional file 6: Fig. S4. Hierarchical clustering and missense mutations for SH2. Top: a dendrogram showing hierarchical clustering of haplotypes across the SH2 gene. The gene is located at position 8,176,778 to 8,183,084: 1,246,231 bases downstream of Cyp6m2. The colour bar indicates the population of origin for each haplotype. Bottom: high frequency (> 5%) alleles identified within each haplotype (white = reference allele; black = alternative allele). [file 12936_2021_3757_MOESM6_ESM.pdf]

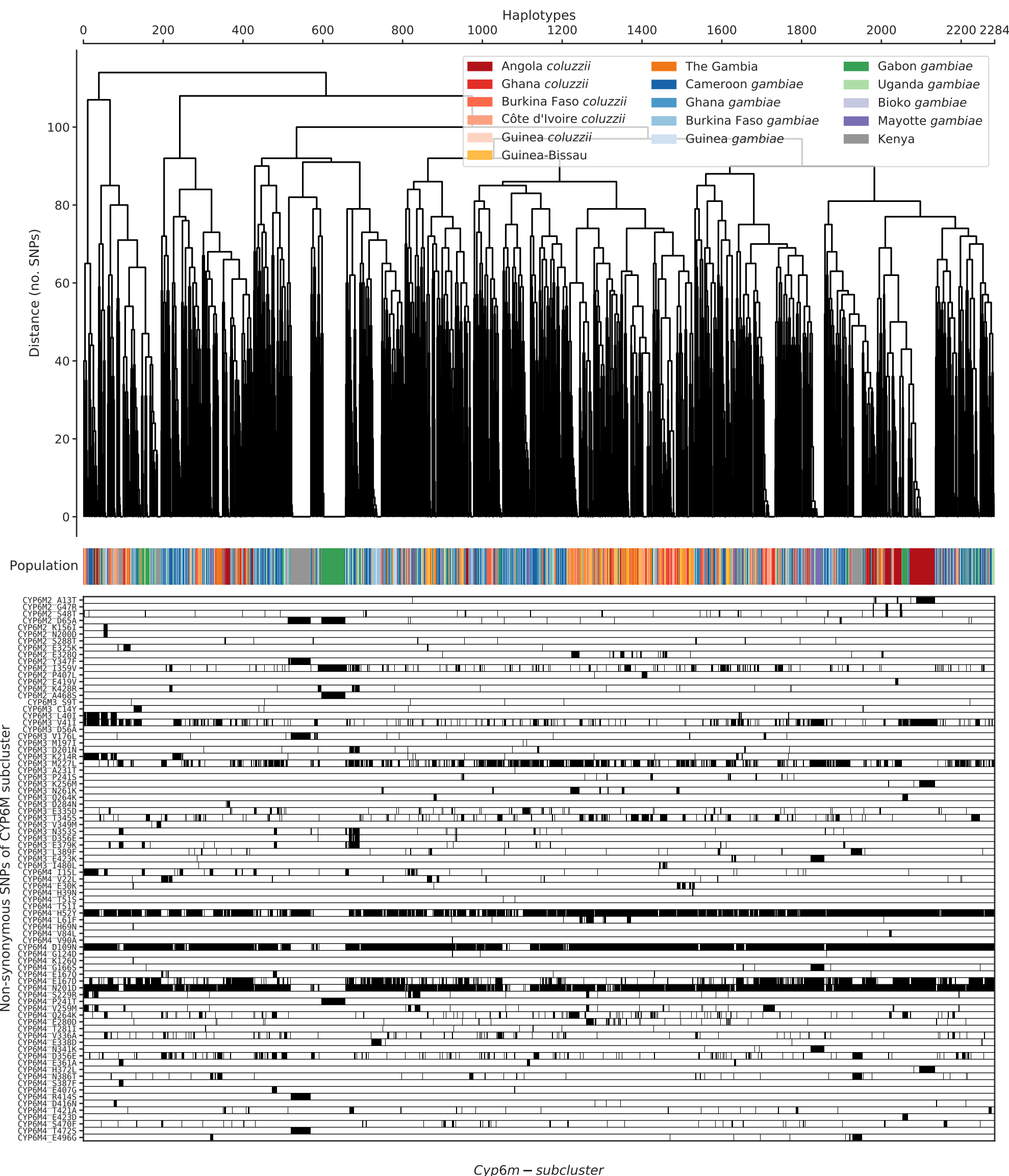

Supplement: Supplementary file 7 — Additional file 7: Fig. S5. Hierarchical clustering and missense mutations for Cyp6m sub cluster. Top: a dendrogram showing hierarchical clustering of haplotypes across the Cyp6m sub cluster of genes containing Cyp6m2, Cyp6m3 and Cyp6m4. The genes are located at position 6928858 to 6935721. The colour bar indicates the population of origin for each haplotype. Bottom: high frequency (> 5%) alleles identified within each haplotype (white = reference allele; black = alternative allele). [file 12936_2021_3757_MOESM7_ESM.pdf]

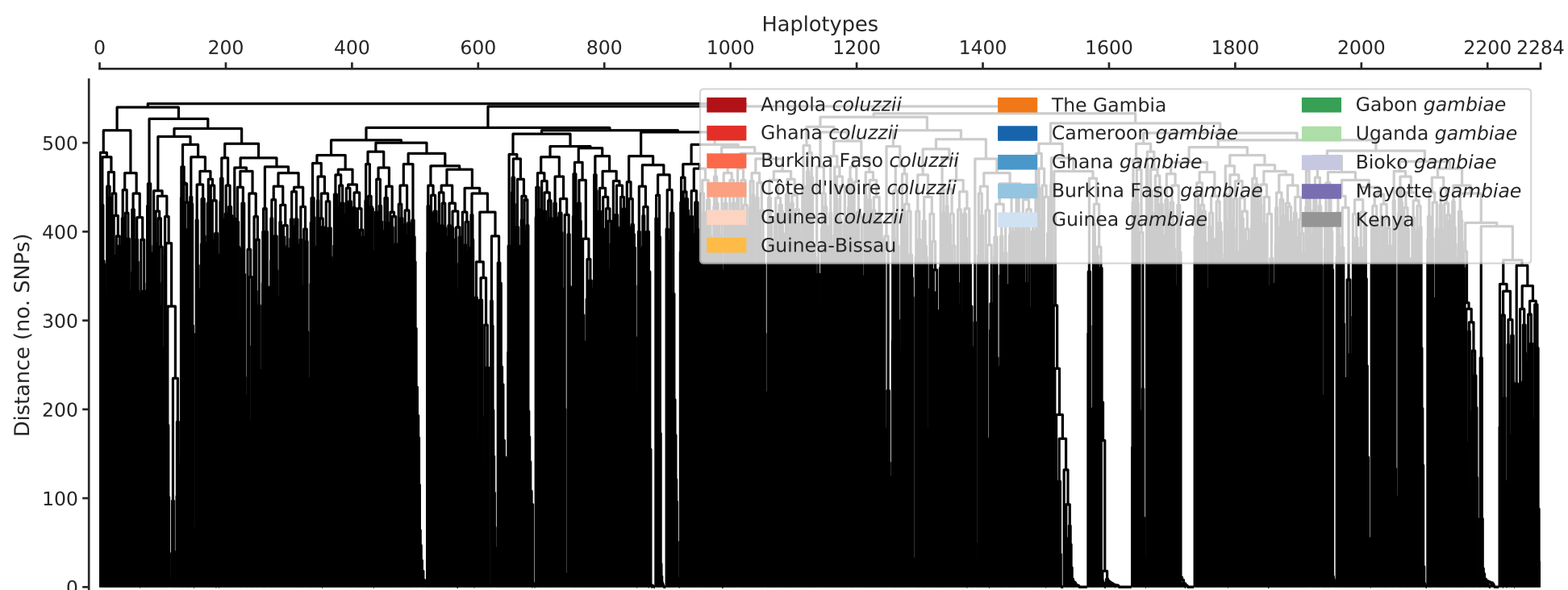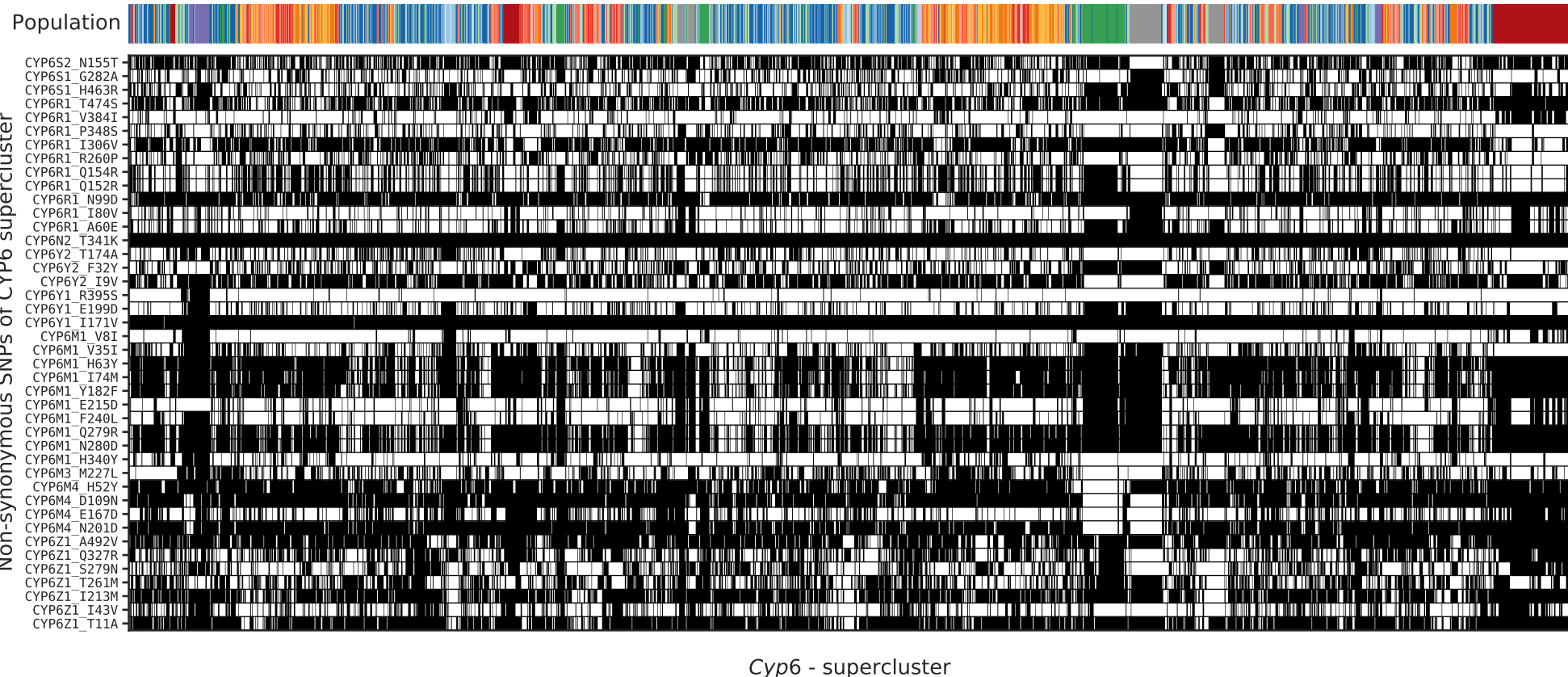

Supplement: Supplementary file 8 — Additional file 8: Fig. S6. Hierarchical clustering and missense mutations for Cyp6 supercluster. Top: a dendrogram showing hierarchical clustering of haplotypes across the Cyp6 supercluster of 14 P450 genes containing Cyp6s2, Cyp6s1, Cyp6r1, Cyp6n2, Cyp6y2, Cyp6y1, Cyp6m1, Cyp6n1, Cyp6m2, Cyp6m3, Cyp6m4, Cyp6z3, Cyp6z2 and Cyp6z1. The genes are located at position 6903106 to 6978142. The colour bar indicates the population of origin for each haplotype. Bottom: high frequency (> 70%) alleles identified within each haplotype (white = reference allele; black = alternative allele). [file 12936_2021_3757_MOESM8_ESM.pdf]

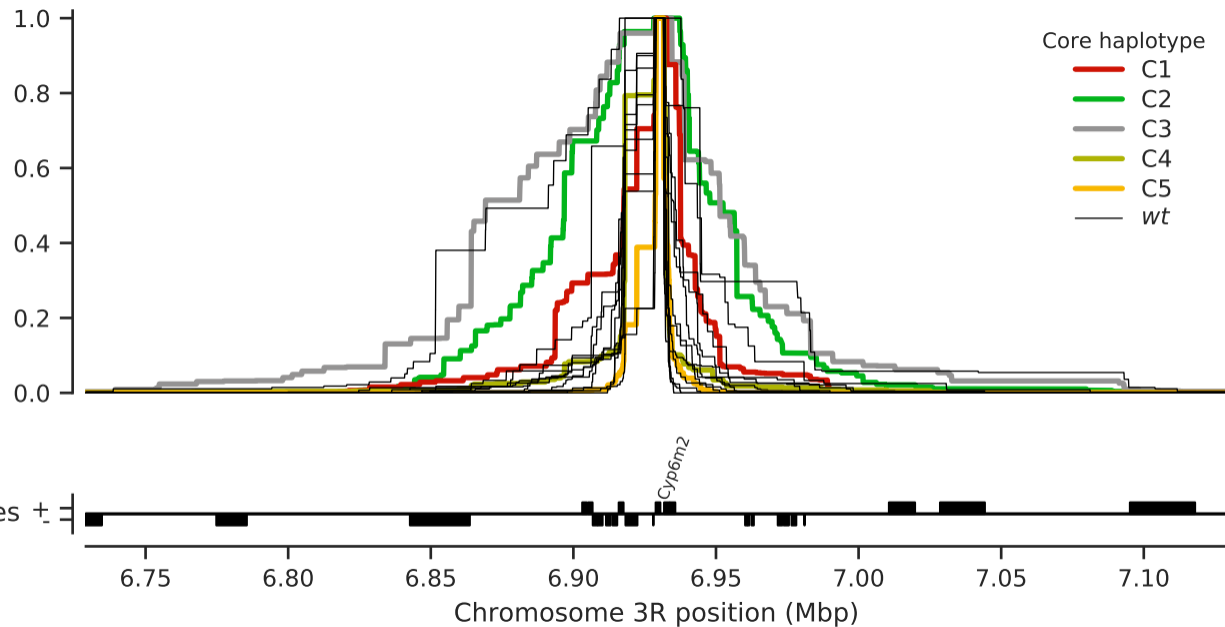

Supplement: Supplementary file 9 — Additional file 9: Fig. S7. Extended haplotype homozygosity across all populations. A rapid decay of EHH in comparison to other haplotypes implies absence of positive selection. [file 12936_2021_3757_MOESM9_ESM.pdf]
